# Polydeoxyribonucleotide, an Adenosine-A2_A_ Receptor Agonist, Preserves Blood Testis Barrier from Cadmium-Induced Injury

**DOI:** 10.3389/fphar.2016.00537

**Published:** 2017-01-10

**Authors:** Francesco Squadrito, Antonio Micali, Mariagrazia Rinaldi, Natasha Irrera, Herbert Marini, Domenico Puzzolo, Antonina Pisani, Cesare Lorenzini, Andrea Valenti, Rosaria Laurà, Antonino Germanà, Alessandra Bitto, Gabriele Pizzino, Giovanni Pallio, Domenica Altavilla, Letteria Minutoli

**Affiliations:** ^1^Department of Clinical and Experimental Medicine, University of Messina Messina, Italy; ^2^Department of Biomedical and Dental Sciences and Morphofunctional Imaging, University of Messina Messina, Italy; ^3^Department of Human Pathology, University of Messina Messina, Italy; ^4^Department of Veterinary Sciences, University of Messina Messina, Italy

**Keywords:** cadmium, PDRN, blood-testis barrier, TGF-β3, pERK 1/2, TUNEL, immunohistochemistry, transmission electron microscopy

## Abstract

Cadmium (Cd) impairs blood-testis barrier (BTB). Polydeoxyribonucleotide (PDRN), an adenosine A_2A_ agonist, has positive effects on male reproductive system. We investigated the effects of PDRN on the morphological and functional changes induced by Cd in mice testes. Adult Swiss mice were divided into four groups: controls administered with 0.9% NaCl (1 ml/kg, i.p., daily) or with PDRN (8 mg/kg, i.p. daily), animals challenged with Cd chloride (CdCl_2_; 2 mg/kg, i.p, daily) and animals challenged with CdCl_2_ (2 mg/kg, i.p., daily) and treated with PDRN (8 mg/kg, i.p., daily). Experiments lasted 14 days. Testes were processed for biochemical, structural, and ultrastructural evaluation and hormones were assayed in serum. CdCl_2_ increased pERK 1/2 expression and Follicle Stimulating Hormone (FSH) and Luteinizing Hormone (LH) levels; it decreased testosterone (TE) and inhibin-B levels and induced structural damages in extratubular compartment and in seminiferous epithelium, with ultrastructural features of BTB disruption. Many TUNEL-positive germ cells were present. CdCl_2_ increased tubular TGF-β3 immunoreactivity and reduced claudin-11, occludin, and *N*-cadherin immunoreactivity. PDRN administration reduced pERK 1/2 expression, FSH, and LH levels; it increased TE and inhibin-B levels, ameliorated germinal epithelium changes and protected BTB ultrastructure. Few TUNEL-positive germ cells were present and the extratubular compartment was preserved. Furthermore, PDRN decreased TGF-β3 immunoreactivity and enhanced claudin-11, occludin, and *N*-cadherin immunoreactivity. We demonstrate a protective effect of PDRN on Cd-induced damages of BTB and suggest that PDRN may play an important role against Cd, particularly against its harmful effects on gametogenesis.

## Introduction

Cadmium (Cd) and its derivative compounds are considered ubiquitous toxicants with carcinogenic activity by the International Agency for Research on Cancer (IARC, 2012). Even if the highest exposure usually takes place in polluted occupational workplaces that utilize Cd (Helmestam et al., 2010), general population is also at risk, owing to the widespread contamination of water and food in exposed sites nearby the factories. In agreement with this assumption, children living in areas at elevated risk showed urinary Cd levels significantly higher than those of control population (Interdonato et al., 2014; Pizzino et al., 2014). Cd was associated with adverse toxic effects in various organs, including the testes (Minutoli et al., 2015b). Indeed, increased incidence of testicular cancer (Pizent et al., 2012), poor semen quality, male infertility (Benoff et al., 2009; Wang et al., 2016) and delayed puberty with reduced gonadal growth (Interdonato et al., 2015) were observed in human subjects exposed to Cd. In animals, Cd was associated with significant decrease in sperm concentration, lower testes and epididymis weight, and evident morphological changes involving sperm and Leydig cells (Acharya et al., 2008; Monsefi et al., 2010).

Blood-testis barrier is crucial for spermatogenesis, as it separates the seminiferous epithelium into basal and adluminal compartments by means of tight (TJs), adherens (AJs), and gap junctions between adjacent Sertoli cells (Singh et al., 2013). Differently from other epithelia, the junctions of the seminiferous epithelium have a single location over the basement membrane. TJs and AJs are made by a transmembrane region, formed by molecules which are present on both adjacent cells (occludin, claudin, junctional adhesion molecules, and *N*-cadherin), linked to other proteins, such as ZO-1, anchored on the inner side to the cytoskeleton (Pérez et al., 2012). During spermatogenesis, spermatogonia undergo the last mitotic division to differentiate into preleptotene spermatocytes; these cells progress to leptotene spermatocytes in the basal compartment, passing through BTB and entering the adluminal compartment (Contuk et al., 2012).

Under normal conditions, BTB function is influenced by a number of factors. Among them, the cytokine TGF-β3, the most abundant form of TGF-β in testis, is produced by Sertoli cells, spermatogonia, and early spermatocytes (Droździk et al., 2015). TGF-β3 mediates its effects on Sertoli cell tight junctions via p38 and pERK 1/2 pathway. An increase in TGF-β3 was paralleled by a decreased content of occludin and ZO-1 in an “*in vivo*” model of Cd-induced infertility (Cheng and Mruk, 2012). In addition, other studies demonstrated that Cd administration induced BTB disruption, affecting the integrity of membrane proteins, such as occludin, ZO-1, *N*-cadherin, and claudin-11, thus causing the release of immature germ cells and interfering with normal spermatogenesis (Xia et al., 2009; Yang et al., 2014; Minutoli et al., 2015b).

An important role in the maintenance of spermatogenesis is played by adenosine. In fact, it was observed in different tissues and organs that the administration of adenosine or the up-regulation of endogenous adenosine (Adair et al., 2005; Valls et al., 2009) was able to protect tissues from inflammatory damage (Campo et al., 2015), through the activation of the adenosine specific cell surface receptor A_2A_ (De Ponti et al., 2007; Minutoli et al., 2011, 2012; Arena et al., 2012).

Polydeoxyribonucleotide was shown to activate the adenosine A_2A_ receptor (Squadrito et al., 2014). PDRN is a compound formed by a mixture of deoxyribonucleotide polymers of different lengths and nucleosides derived from salmon trout (*Oncorhynchus mykiss*) sperm by a process that guarantees a high percentage of DNA and the absence of active proteins or peptides (Guizzardi et al., 2003). PDRN effects were documented in several experimental models (Lazzarotto et al., 2004; Guizzardi et al., 2007; Bitto et al., 2008; Galeano et al., 2008; Altavilla et al., 2009; Kim et al., 2014). Furthermore, as adenosine and its analogs, it can stimulate sperm functional ability in mouse (Fraser and Duncan, 1993). Interestingly, PDRN was also tested in rats after experimental varicocele or testis ischemia/reperfusion injury (I/R). In fact, in experimental varicocele (Minutoli et al., 2011, 2015a; Arena et al., 2012), PDRN induced Vascular Endothelial Growth Factor-A (VEGF-A) production, thus promoting intratesticular vascularization and improving spermatogenesis. In testis after I/R injury (Minutoli et al., 2012), PDRN increased VEGF-A and endothelial Nitri**c** Oxide Synthase (eNOS), with positive effects on spermatogenesis.

In light of the above observations, we investigated the effects of PDRN in mice exposed to Cd chloride (CdCl_2_) to evaluate the role of the adenosine agonist on BTB integrity. On the basis of the achieved results, PDRN is proposed as a potential therapeutic tool for human sub- or infertility induced by exposition to environmental toxicants, such as Cd.

## Materials and Methods

### Experimental Protocol

All procedures complied with the standards for care and use of animal subjects as stated in the Guide for the care and use of laboratory animals (Institute of Laboratory Animal Resources, National Academy of Sciences, Bethesda, MD, USA). Sixty-four male adult C57 BL/6J mice (25–30 g) were purchased from Charles River Laboratories Italia srl (Calco, Italy). The animals were provided a standard diet *ad libitum* with free access to tap water and were maintained on a 12-h light/dark cycle. The animals were divided into four groups: (i) animals administered with a vehicle solution consisting in 0.9% NaCl (1 ml/kg, i.p., daily), named “control + vehicle animals,” (ii) animals administered with PDRN (8 mg/kg, i.p. daily) named “control + PDRN animals,” (iii) animals challenged with CdCl_2_ plus with the same vehicle as above (2 mg/kg, i.p., daily), named “CdCl_2_ + vehicle animals”; and (iv) animals challenged with CdCl_2_ (2 mg/kg, i.p., daily) and treated with PDRN (8 mg/kg, i.p., daily), immediately after CdCl_2_ administration, named “CdCl_2_ + PDRN animals.” From each group, four animals were used for histological evaluation with Trypan blue (16 animals), five animals were used for transmission electron microscopy (20 animals), seven animals were used for all the other procedures (28 animals). The experiments lasted 14 days until mice were sacrificed with an overdose of ketamine and xylazine (100/20 mg/kg, i.p., respectively) and then subjected to bilateral orchidectomy. Explanted testes were then processed for biochemical, structural, immunohistochemical, and ultrastructural analyses.

### Western Blot Analysis

To extract total cellular proteins, tissue samples were treated one time, at 4°C, with a lysis buffer composed by 25 mM Tris-HCl pH 7.4, 1.0 mM EGTA, 1.0 mM EDTA, 0.5 mM phenylmethylsulfonyl fluoride, added with protease and phosphatase inhibitors [100 mM Na_3_VO_4_, aprotinin, leupeptin, pepstatin (10 μg/ml each)]. The cell lysate was centrifuged at 13000 rpm for 15 min and the supernatant was used for protein concentration determination by Bio-Rad protein assay (Bio-Rad, Richmond, CA, USA) and then diluted with Laemmli buffer. Protein samples were denatured in reducing buffer (62 mM Tris-HCl pH 6.8, 10% glycerol, 2% SDS, 5% beta-mercaptoethanol, 0.003% bromophenol blue) and separated by electrophoresis on SDS polyacrylamide gel (6% or 10%), approximately for 1 h. The separated proteins were transferred to a PVDF membrane in a transfer buffer [39 mM glycine, 48 mM Tris-HCl (pH 8.3), 20% methanol] at 200 mA for 1 h. The membranes were then blocked with 5% non-fat dry milk in TBS-0.1% Tween-20 for 1 h at room temperature. Membranes were washed three times for 10 min each in TBS-0.1% Tween-20 and incubated with a primary antibody for pERK 1/2 (Cell Signaling, Beverly, MA, USA) diluted 1:500 in TBS-0.1% Tween-20 overnight at 4°C. The day after the membranes were washed three times for 10 min in TBS-0.1% Tween-20 and were incubated with a specific peroxidase-conjugated secondary antibody (1:10,000; KPL, USA) for 1 h at room temperature. Following other washings, the membranes were analyzed by enhanced chemiluminescence (KPL, USA). Protein signals were quantified by scanning densitometry using a bio-image analysis system (C-DiGit Blot Scanner with Image Studio software) and the results were expressed as relative integrated intensity compared to controls. β-actin (Cell Signaling Technology, Beverly, MA, USA) was used to confirm equal protein loading and blotting.

### Hormone Levels

Follicle-stimulating hormone, LH, TE, and inhibin B were studied in serum by ELISA. The samples were analyzed in duplicate, following carefully the protocols suggested by manufacturers. Briefly, blood was obtained from cardiac puncture and serum was achieved by centrifugation for 10 min at 1000 × *g*. An HRP-conjugate and the specific antibody were added. After two washes with buffer, substrates were added, followed by stop solution. The mean absorbances were calculated using a microplate reader at 450 nm and correlated with those from standard curves. Data were expressed in mIU/ml for FSH and LH, in ng/ml for TE, and in pg/ml for inhibin B.

### Histological Evaluation

The testes of the four groups mice were fixed in 4% paraformaldehyde in 0.2 M phosphate buffer saline (PBS), dehydrated in graded ethanol, cleared in xylene and embedded in Paraplast (SPI Supplies, West Chester, PA, USA). Five micrometers sections were stained with hematoxylin and eosin (H&E) and photographed with a Zeiss Primo Star (Carl Zeiss Inc, Oberkochen, Germany) light microscope. Images were taken with a Canon A620 Powershot camera and blindly examined by two trained observers without knowledge of the previous treatment. Five microscopic fields from ten non-serial sections of each group were considered. Both tubular and extratubular compartments were considered for the morphological evaluation. In the tubular compartment, the diameters of 100 separate seminiferous tubules, all showing a circular profile, were measured to calculate the mean seminiferous tubule diameter (MSTD). A Peak Scale Loupe 7x (GWJ Company, Hacienda Heights, CA, USA) micrometer was used as a scale calibration standard to calculate the diameters, expressed in micrometers (μm). Spermatogenesis was quantified with Johnsen’s scoring system (Johnsen, 1970), as modified by Erdemir et al. (2012). In the extratubular compartment, edema, hemorrhagic extravasation, venular and/or lymphatic vessels dilation, and Leydig cells changes were evaluated according to a previously described method (Minutoli et al., 2005, 2015a), scoring each parameter with the following scale: 0, absent; 1, mild; 2, moderate; 3, severe.

### Histological Evaluation with Trypan Blue

Four mice from control animals administered with 0.9% NaCl, four from control animals administered with PDRN, four from CdCl_2_ challenged group, and four from CdCl_2_ plus PDRN challenged group received an i.p. injection of 0.5 ml of 3% Trypan blue in saline 48 h before sacrifice, to label testicular macrophages (Miller et al., 1983; Gaytan et al., 1994). The testes were then processed as above indicated for histological evaluation, being sections counterstained only with hematoxylin.

### Terminal Deoxynucleotidyl Transferase Enzyme Mediated dUTP Nick End Labeling (TUNEL) Immunohistochemistry

For TUNEL technique, a Universal Apoptosis Detection Kit (GenScript, Piscataway, NJ, USA) was used. On 5 μm sections, after protein digestion with proteinase K (20 μg/mL PBS), the activity of endogenous peroxidase was stopped with 3% H_2_O_2_ in methanol. Sections were incubated with terminal deoxynucleotidyl transferase enzyme and biotin-11-dUTP, with streptavidin-peroxidase substrate, and with 3,3′-diaminobenzidine tetrahydrochloride. Specimens were evaluated as previously indicated for histological sections. From each group, the percentage of tubules with apoptotic cells (%TWAC) and the apoptotic index, indicating the mean number of TUNEL-positive cells per tubule (Tsounapi et al., 2012), were calculated.

### Immunohistochemistry for Claudin-11, Occludin, *N*-Cadherin, and TGF-β3

Histological sections (5 μm) were deparaffinized in xylene and rehydrated in 100, 95, 80, and 70% ethanol. Antigen retrieval was performed with pH 6.0 buffer citrate and endogenous peroxidase blocking with 0.3 % H_2_O_2_ in PBS. Sections were incubated overnight in 1% bovine serum albumin in PBS at room temperature. Primary antibodies (claudin-11 and *N*-cadherin, both 1/200 dilution: Santa Cruz, Dallas, USA; occludin and TGF-β3, 1/50 and 1/100 dilution, respectively: Abcam, Cambridge, UK) were incubated overnight at 4°C in a moisturized chamber and the day after peroxidase-conjugated secondary antibody (1/50 dilution; Pierce anti-rabbit, anti-goat, and anti-mouse, Cambridge, UK) was added and reaction visualized with 3,3′-Diaminobenzidine (Sigma–Aldrich, Milan, Italy). Counterstaining was performed with haematoxylin alone. Negative control slices were tested using PBS instead of primary antibody.

### Transmission Electron Microscopy (TEM)

The testes of five mice from control animals administered with 0.9% NaCl, five from control animals administered with PDRN, five from CdCl_2_ challenged group, and five from CdCl_2_ plus PDRN challenged group were fixed by immersion in 2.5% glutaraldehyde in 0.1 M phosphate buffer (pH 7.4) at +4°C, washed with 0.1 M phosphate buffer (pH 7.4), post-fixed in 1% OsO_4_ in 0.2 M phosphate buffer (pH 7.4) at +4°C for 1h, dehydrated in graded ethanol, immersed in propylene oxide, and embedded in Durcupan (Sigma–Aldrich/Fluka, St. Louis, MO, USA). Ultrathin silver-golden sections were cut with a diamond knife on a Reichert Jung Ultracut E, placed on uncoated 200 mesh copper grids, contrasted with methanolic uranyl acetate and lead citrate (Reynolds, 1963) and photographed with a JEOL JEM-100 SX transmission electron microscope at 80 kV.

### Drugs

CdCl_2_ was purchased from Sigma–Aldrich Srl (Milan, Italy) and dissolved in 0.9% NaCl. PDRN was a kind gift from Mastelli srl, Sanremo, Italy. All chemicals and reagents were commercially available reagent grades.

### Statistical Analysis

Values are provided as mean ± standard error (SE). The statistical significance of differences among groups was performed with ANOVA comparison tests. Mann–Whitney *U* tests with Bonferroni correction was used for the statistical analysis of histological scores. A *p* value ≤ 0.05 was considered statistically significant.

## Results

### PDRN Decreases pERK 1/2 Expression

**FIGURE 1:**
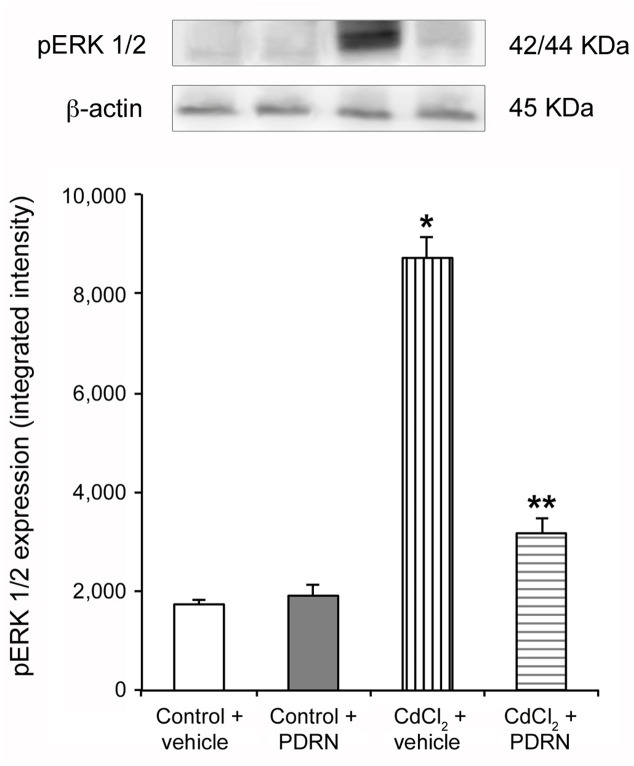
**Representative Western Blot analysis of p-ERK 1/2 of testis-derived lysates obtained from the two types of control animals (Control plus vehicle; Control plus PDRN) and the two types of treated ones (CdCl_2_ plus vehicle; CdCl_2_ plus PDRN)**. β-actin was used as an internal reference. ^∗^*p* < 0.05 vs. controls; ^∗∗^*p* < 0.05 vs. CdCl_2_. Bars represent the mean ± SE of seven experiments.

Low expression of pERK 1/2 was detected in testes of control mice treated with either vehicle or PDRN (**Figure 1**). An increased expression of pERK 1/2 was observed in CdCl_2_ challenged animals (**Figure 1**). PDRN administration significantly reduced pERK 1/2 expression when compared to CdCl_2_ alone mice (**Figure 1**).

### PDRN Restores the Hormonal Status

**FIGURE 2:**
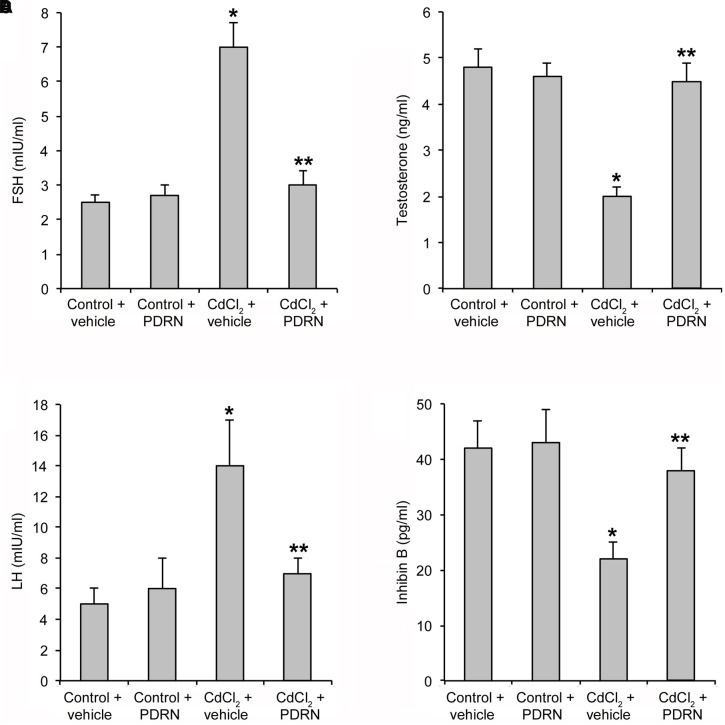
**Levels of FSH (A)**, TE **(B)**, LH **(C)**, and inhibin B **(D)** in testes collected from control plus vehicle, control plus PDRN (8 mg/kg i.p.), CdCl_2_ (2 mg/kg/day i.p.) plus vehicle, and CdCl_2_ (2 mg/kg/day i.p.) plus PDRN (8 mg/kg i.p.) treated mice. ^∗^*p* < 0.05 vs. controls; ^∗∗^*p* < 0.05 vs. CdCl_2_ plus vehicle. Bars represent the mean ± SE of seven experiments.

Control mice treated with either vehicle or PDRN had normal serum levels of FSH, LH, TE, and inhibin B (**Figures 2A–D**). CdCl_2_ challenge significantly enhanced FSH and LH levels and reduced TE and inhibin B serum concentration (**Figures 2A–D**). PDRN restored the hormonal status of CdCl_2_ challenged animals, being the levels of FSH, LH, TE, and inhibin B not significantly different that those of both control groups of mice (**Figures 2A–D**).

### PDRN Preserves Morphological Features

**FIGURE 3:**
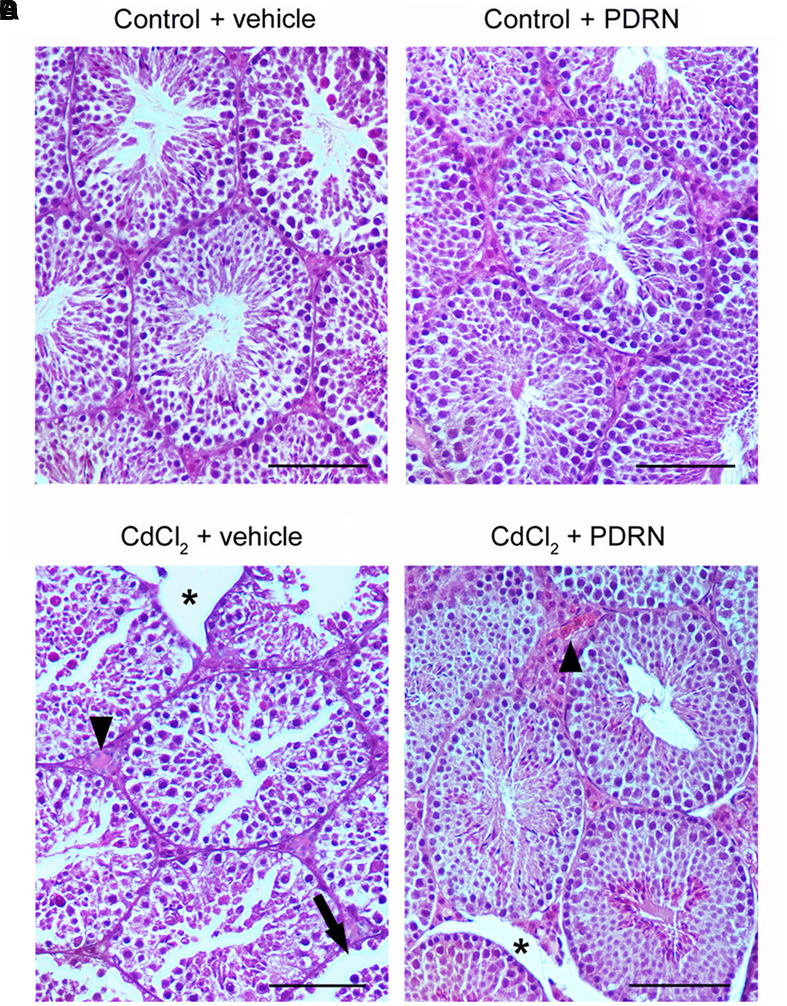
**Representative histological sections of a testis from a control plus vehicle animal (A)**, a control plus PDRN animal **(B)**, a CdCl_2_ (2 mg/kg i.p.) plus vehicle animal **(C)**, and a CdCl_2_ (2 mg/kg i.p.) plus PDRN (8 mg/kg i.p.) animal **(D)**. **(A,B)** Seminiferous tubules and extratubular compartment show normal morphology. **(C)** Arrow, Round spermatids and spermatogonia detached from the basal membrane; ^∗^, marked edema of the extratubular compartment; Arrowhead, hemorrhagic extravasation. **(D)** ^∗^, mild interstitial edema; Arrowhead, enlarged vessels in the extratubular compartment. (Scale bar: 50 μm).

The seminiferous epithelium and the extra tubular compartment exhibited normal features for both control groups (**Figures 3A,B**; **Tables 1** and **2**). By contrast, in testes of mice challenged with CdCl_2_ the germinal epithelium showed a low Johnsen’s score, some round spermatids and marked detachments from the basal membrane of both Sertoli cells and spermatogonia. An evident hemorrhagic extravasation and an interstitial edema were apparent in the extratubular compartment (**Figure 3C**; **Tables 1** and **2**). In mice challenged with CdCl_2_ and treated with PDRN, the seminiferous tubules had larger size and the germinal epithelium was better preserved, with a higher Johnsen’s score and many spermatozoa. However, milder edema and hemorrhagic extravasation were still present in the extratubular compartment (**Figure 3D**; **Tables 1** and **2**).

**Table 1:** Effects on testis tubular compartment for the two types of control animals (Control plus vehicle; Control plus PDRN) and the two types of treated ones (CdCl_2_ plus vehicle; CdCl_2_ plus PDRN).

	Tubular compartment			
	Mean Seminiferous Tubule Diameter (μm)	Johnsen’s score	% TWAC	Apoptotic index
Control plus vehicle	177.6 ± 8.5	9.6 ± 0.6	2	0.2 ± 0.3
Control plus PDRN	175.4 ± 7.8	9.4 ± 0.4	3	0.3 ± 0.3
CdCl_2_ plus vehicle	139.2 ± 13.5^a^	5.2 ± 0.7^a^	38^a^	7.9 ± 1.1^a^
CdCl_2_ plus PDRN	163.6 ± 6.6^b^	7.4 ± 0.2^b^	8^b^	1.2 ± 1.1^b^

^a^*p < 0.05 vs. control; ^b^*p* < 0.05 vs. CdCl_2_ plus vehicle*.

**Table 2:** Effects on testis extratubular compartment for the two types of control animals (Control plus vehicle; Control plus PDRN) and the two types of treated ones (CdCl_2_ plus vehicle; CdCl_2_ plus PDRN).

	Extratubular compartment			
	Edema	Hemorrhagic extravasation	Vascular dilation	Leydig cells changes
Control plus vehicle	0	0	0	0
Control plus PDRN	0	0	0	0
CdCl_2_ plus vehicle	1.4 ± 0.3^a^	1.8 ± 0.3^a^	1.7 ± 0.4^a^	1.3 ± 0.2^a^
CdCl_2_ plus PDRN	0.7 ± 0.3^b^	0.8 ± 0.3^b^	0.7 ± 0.3^b^	0.4 ± 0.3^b^

^a^*p < 0.05 vs. control; ^b^*p* < 0.05 vs. CdCl_2_ plus vehicle. Histologic grading of the extratubular compartment changes was based on the following scale: 0, absent; 1, mild; 2, moderate; 3, severe*.

### PDRN Modulates Apoptotic Pattern

**FIGURE 4:**
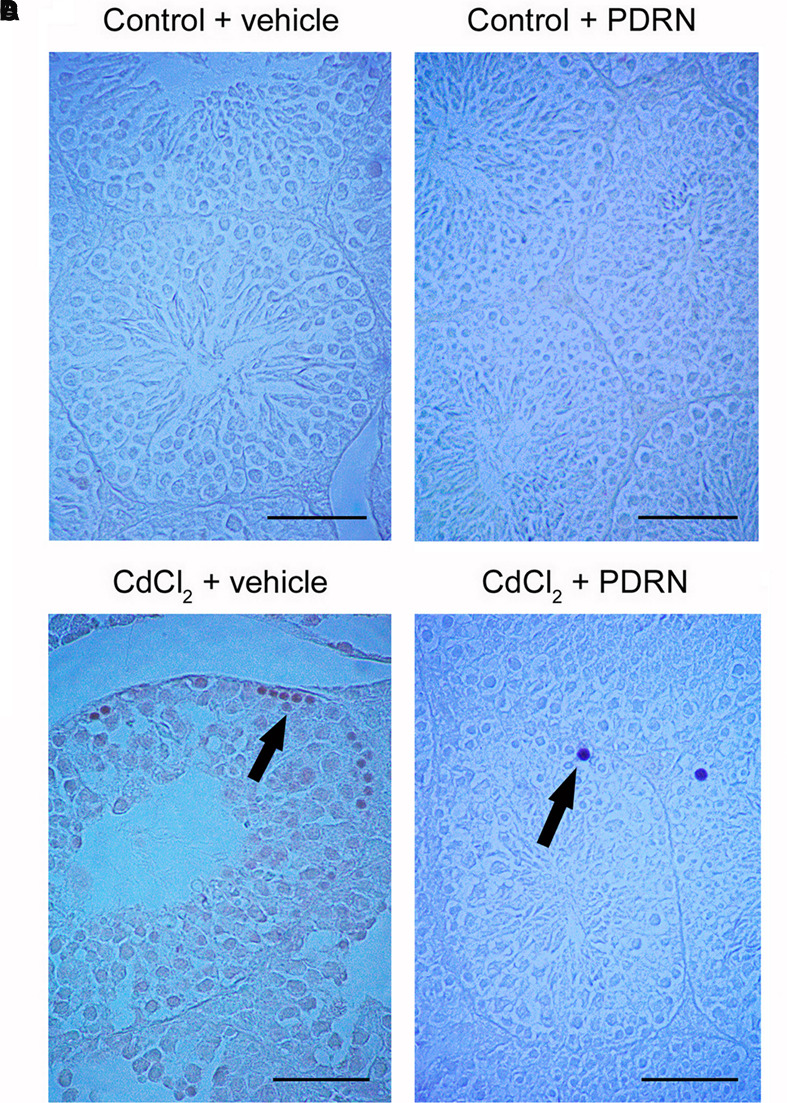
**TUNEL staining technique revealing apoptosis in the testes from control plus vehicle animals (A)**, control plus PDRN (8 mg/kg i.p.) animals **(B)**, CdCl_2_ (2 mg/kg i.p.) plus vehicle animals **(C)**, and CdCl_2_ (2 mg/kg i.p.) plus PDRN (8 mg/kg i.p.) animals **(D)**. **(A,B)** No TUNEL-positive cells are present. **(C)** Arrow, peripheral clusters of TUNEL-positive cells. **(D)** Arrow, isolated TUNEL-positive cells. (Scale bar: 50 μm).

No TUNEL-positive germ cells were present in the seminiferous tubules from both control groups (**Figures 4A,B**; **Table 1**). On the contrary, after challenge with CdCl_2_ a lot of TUNEL-positive germ cells were grouped along small peripheral districts of the seminiferous tubules (**Figure 4C**; **Table 1**). In testes of CdCl_2_ mice treated with PDRN, few isolated peripheral positive germ cells were observed (**Figure 4D**; **Table 1**).

### PDRN Controls Macrophage Migration

**FIGURE 5:**
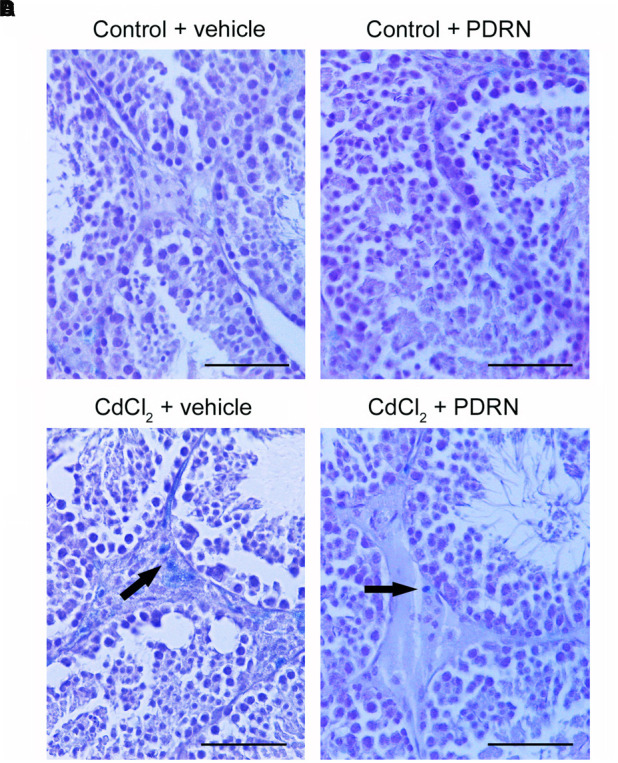
**Trypan blue staining technique revealing interstitial macrophages in the testes from control plus vehicle animals (A)**, control plus PDRN (8 mg/kg i.p.) animals **(B)**, CdCl_2_ (2 mg/kg i.p.) plus vehicle animals **(C)**, and CdCl_2_ (2 mg/kg i.p.) plus PDRN (8 mg/kg i.p.) animals **(D)**. **(A,B)** No macrophages are present in the extratubular compartment. **(C)** Arrow, numerous interstitial macrophages. **(D)** Arrow, isolated interstitial macrophages. (Scale bar: 80 μm)

Testes from both control groups showed no macrophages in the extratubular compartment, as evaluated by means of Trypan blue staining (**Figures 5A,B**). By contrast, many macrophages were evident in the extratubular spaces of CdCl_2_ challenged mice (**Figure 5C**). On the contrary, in CdCl_2_ mice treated with PDRN few isolated macrophages were present in the extratubular compartment (**Figure 5D**).

### PDRN Regulates TGF-β3, Claudin-11, Occludin, and *N*-Cadherin Immunoreactivity

**FIGURE 6:**
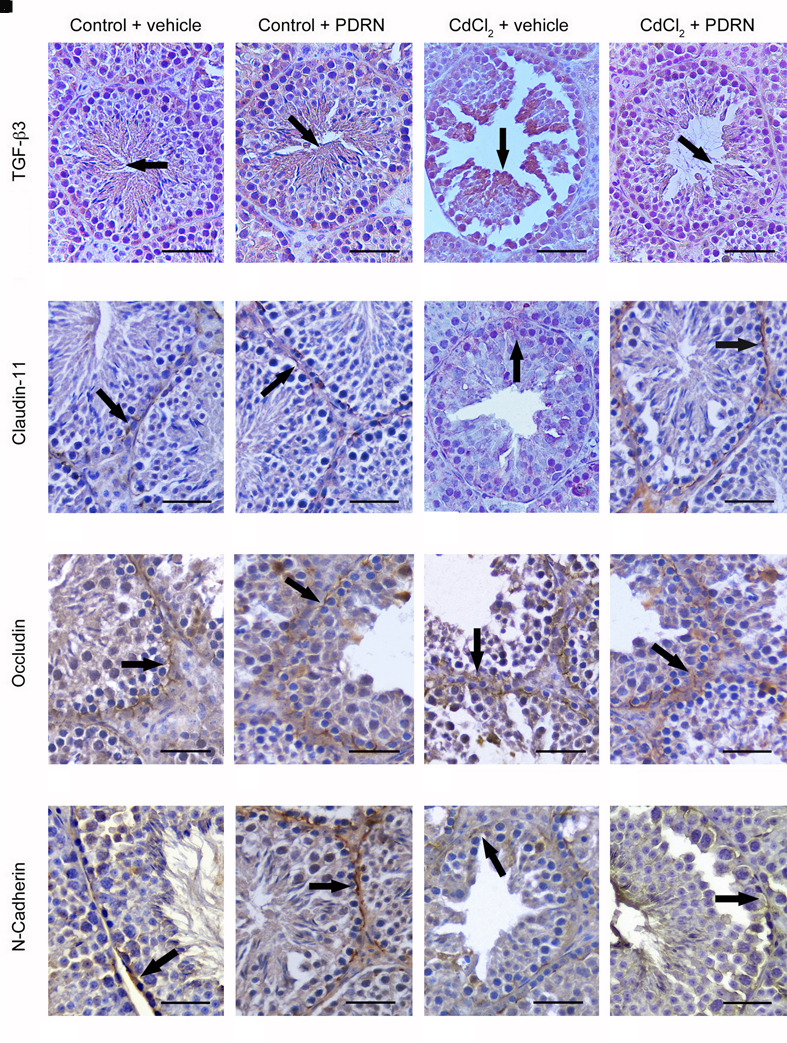
**Immunohistochemical localization of TGF-β3 (A–D)**, claudin-11 **(E–H)**, occludin **(I–L)**, and *N*-cadherin **(M–P)** in the testes from control plus vehicle animals **(A,E,I,M)**, control plus PDRN (8 mg/kg i.p.) animals **(B,F,J,N)**, CdCl2 (2 mg/kg i.p.) plus vehicle animals **(C,G,K,O)**, and CdCl2 (2 mg/kg i.p.) plus PDRN (8 mg/kg i.p.) animals **(D,H,L,P)**. **(A,B)** Arrow, TGF-β3 adluminal immunoreactivity. **(C)** Arrow, increased TGF-β3 reactivity of round spermatids. **(D)** Arrow, weak TGF-β3 adluminal immunoreactivity. **(E,F)** Arrow, normal basal claudin-11 immunoreactivity. **(G)** Arrow, weak and irregular claudin-11 immunoreactivity. **(H)** Arrow, basal claudin-11 immunoreactivity. **(I,J)** Arrow, normal basal occludin immunoreactivity. **(K)** Arrow, diffuse, irregular occludin immunoreactivity. **(L)** Arrow, occludin immunoreactivity along the basal compartment. **(M,N)** Arrow, *N*-cadherin immunoreactivity in the basal part of seminiferous epithelium. **(O)** Arrow, irregular basal positivity for *N*-cadherin. **(P)** Arrow, basal immunoreactivity for *N*-cadherin. (Scale bar: 50 μm)

Round and elongated spermatids, positive for TGF-β3, were present in the tubule adluminal compartment for both groups of control animals (**Figures 6A,B**). Immunoreactivity was markedly increased in round spermatids of CdCl_2_ challenged mice (**Figure 6C**). CdCl_2_ challenged animals treated with PDRN had reduced immunoreactivity in the seminiferous epithelium (**Figure 6D**). An evident immunoreactivity for Claudin-11 of the basal compartment resulted for both groups of control animals (**Figures 6E,F**). In CdCl_2_ challenged mice, immunoreactivity was weak and irregular (**Figure 6G**). The seminiferous tubules of CdCl_2_ plus PDRN animals showed evident immunoreactivity at level of the intercellular junctions of the tubule basal compartment (**Figure 6H**). Occludin immunoreactivity was localized at the intercellular junctions along the basal compartment in both groups of control animals (**Figures 6I,J**). CdCl_2_ challenged mice showed an irregular immunoreactivity (**Figure 6K**). CdCl_2_ injected animals and treated with PDRN had an evident immunoreactivity along the intercellular junctions of the basal compartment of the tubules (**Figure 6L**). *N*-cadherin immunoreactivity was evident in the basal part of seminiferous epithelium and along some Sertoli cells for both groups of control animals (**Figures 6M,N**). CdCl_2_ challenged mice showed reduced and irregular positivity along the basal compartment (**Figure 6O**). By contrast, CdCl_2_ plus PDRN animals showed an evident immunoreactivity in the basal compartment of the tubules (**Figure 6P**).

### PDRN Protects BTB Ultrastructure

**FIGURE 7:**
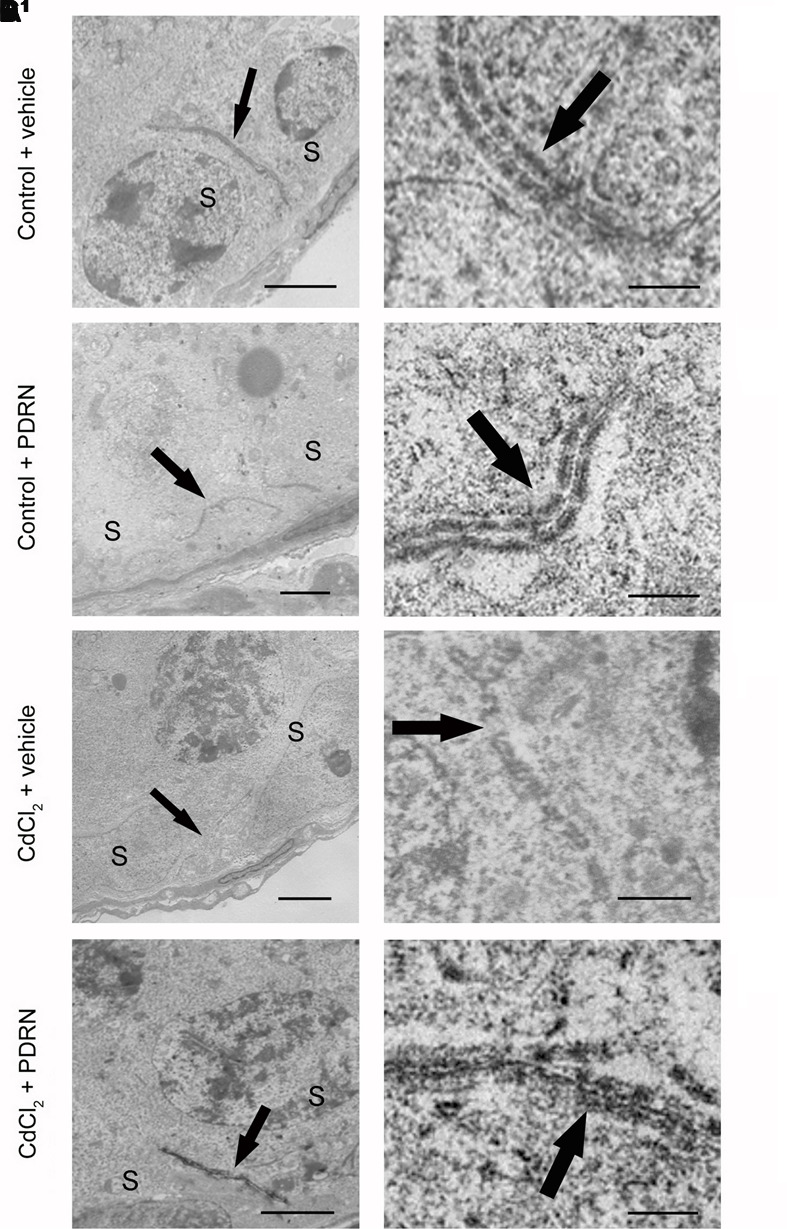
**Transmission electron micrographs of testes from control plus vehicle animals (A,A^1^)**, control plus PDRN (8 mg/kg i.p.) animals **(B,B^1^)**, CdCl_2_ (2 mg/kg i.p.) plus vehicle animals **(C,C^1^)**, and CdCl_2_ (2 mg/kg i.p.) plus PDRN (8 mg/kg i.p.) animals **(D,D^1^)**. **(A,A^1^,B,B^1^)** Arrow, linear junctions between adjacent Sertoli cells (S). **(C,C^1^)** Arrow, fragmented junctions between adjacent Sertoli cells (S). **(D,D^1^)** Arrow, continuous junctions between adjacent Sertoli cells (S). [Scale bar: **(A–D)** = 4 μm; **(A^1^,B^1^)** = 0.5 μm; (**C^1^)** = 1 μm; **(D^1^)** = 0.3 μm).

When observed with TEM, testes from both groups of control animals showed linear and regular junctions sealing two adjacent Sertoli cells (**Figures 7A,A^1^,B,B^1^**). By contrast, fragmented junctions were evident between adjacent Sertoli cells in CdCl_2_ challenged mice (**Figures 7C,C**^1^). Continuous junctions were observed between adjacent Sertoli cells in CdCl_2_ challenged mice administered with PDRN (**Figures 7D,D**^1^).

## Discussion

In adult mammalian testis, the seminiferous epithelium is formed by Sertoli cells and by germ cells. Sertoli cells are large, somatic cells, contacting the basal lamina of the seminiferous tubules and extending to the lumen of tubules (Holstein et al., 2003). Germ cells comprise a stem cell population of basal spermatogonia and a migrating population undergoing the morphological and functional differentiation called spermatogenesis. Both types of germ cells are in close contact with Sertoli cells, which provide a trophic and physical support. Furthermore, adjacent Sertoli cells are connected by intercellular junctions, which form BTB (Gerber et al., 2016): In this way, two distinct compartments are created in the tubules, the basal and the adluminal compartments. In order to allow germ cells to move from the basal to the adluminal compartment, BTB junctions rapidly disassemble and reassemble in a very dynamic way. BTB restructuring allows germ cells progression to the adluminal compartment and involves peculiar changes in structural proteins of tight junctions, such as claudin-11 and occludin, and of adherens junctions, such as *N*-cadherin (Mruk et al., 2014).

Cadmium is a non-essential heavy metal present in the environment at low levels, but a widespread contamination of water and food due to anthropogenic activities has greatly increased its concentration in the atmosphere (Spiazzi et al., 2013). Cd may accumulate in different organs, such as liver, kidneys, lungs, and testes (Haouem et al., 2008), thus inducing peculiar functional and morphological changes. When testes are exposed to Cd, BTB integrity is compromised in response to a reduction of the steady-state levels of integral membrane proteins such as occludin, *N*-cadherin, and claudin-11 (Siu et al., 2009; Yang et al., 2014; Minutoli et al., 2015b). These changes might depend on an enhanced production of TGF-β3, which activates the p38 MAPK signaling pathway downstream (Lui et al., 2003).

Transforming growth factor-β3is a master regulator of BTB, as it controls Sertoli cell junction dynamics (Xia and Cheng, 2005) by guiding the opening and closing of junctions between Sertoli cells and germ cells migrating from the basal to the adluminal compartment (Zhang et al., 2004). In this way, fully developed spermatozoa can be released into the tubular lumen at the end of spermiogenesis (Lui et al., 2003; Mruk et al., 2014).

Our results, in agreement with previous studies (Lui et al., 2003; Siu et al., 2009; Minutoli et al., 2015b), indicate that the immunohistochemical localization of both TGF-β3 and integral proteins occludin, *N*-cadherin and claudin-11 was markedly impaired in Cd challenged mice when compared to normal controls. PDRN administration preserved the morphological and functional aspects of BTB. In addition, the ability of PDRN in maintaining BTB integrity was also supported by the presence of reduced numbers of interstitial macrophages, as revealed by Trypan blue technique (Miller et al., 1983; Gaytan et al., 1994). These effects would be due to a PDRN induced reduction in TGF-β3 synthesis. Indeed, activation of the adenosine specific cell surface receptor A_2A_ has been shown to modulate several growth factors (De Ponti et al., 2007; Minutoli et al., 2011, 2012; Arena et al., 2012).

Previous studies demonstrated that Cd increases the expression of MAPK pERK 1/2 (Ji et al., 2015; Minutoli et al., 2015b), which was considered one of the downstream signal transducers involved in the disassembly of focal adhesion-like ectoplasmic specialization structures in the adluminal compartment of seminiferous tubules (Wong and Cheng, 2005). Furthermore, it is known that other MAPK family components (p38 and JNK) are also able to regulate BTB through TGF-β3 (Lui et al., 2003). Therefore, we evaluated pERK 1/2 expression. PDRN administration significantly reduced the kinase, thus indicating a strong correlation between TGF-β3 and the MAPK component pERK 1/2 to exist. In agreement with immunohistochemical and molecular data, transmission electron micrographs confirmed that PDRN administration was able to maintain an adequate ultrastructural organization of BTB, as shown by the presence of well-evident, continuous junctions between adjacent Sertoli cells. The activation of MAPK pathway induced by Cd challenge is also able to trigger cell apoptosis (Wong and Cheng, 2011). The present work confirms previous experimental data from our laboratory (Minutoli et al., 2015b), as well as those reported in another paper (Khanna et al., 2011). In fact, TUNEL-positive cells were highly reduced in the seminiferous epithelium in CdCl_2_ challenged mice administered with PDRN.

Furthermore, Cd induces negative effects on the structural organization of the interstitial compartment and of the seminiferous tubules. PDRN reduced the extratubular compartment volume, as indicated by a less evident interstitial edema and hemorrhagic extravasation, and the mean tubular diameter and significantly increased Johnsen’s score, thus showing that the A_2A_ agonist had a positive action on spermatogenesis. As to the involved mechanisms, it can be speculated that PDRN, as previously shown for an experimental model of varicocele (Minutoli et al., 2011), activates the A_2A_ receptors of sperm cells and stimulates adenylate cyclase activity (Fraser and Duncan, 1993), acting *in vivo* as a regulator of sperm function even during Cd challenge.

**FIGURE 8:**
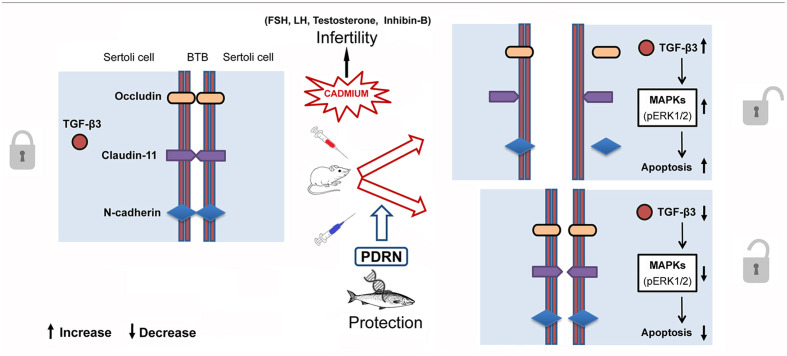
**Schematic representation of the suggested action mechanism of Cd and PDRN through the pERK 1/2/TGF-β3 signaling pathway at level of Sertoli cells in mice**.

The spermatogenetic process is known to be regulated by several hormones including TE, FSH, LH, and inhibin-B (Spiazzi et al., 2013; Mruk et al., 2014). Cd has been shown to affect the hypothalamic-pituitary-gonadal axis, with subsequent disregulation of germ cell differentiation (Meng et al., 2005). In the present work, PDRN administration restored the hormonal status with positive effects on spermatogenesis and Sertoli cells, as also demonstrated by the structural features exhibited by BTB. Thus, it can be argued that this compound may be also able to counteract Cd-induced endocrine disrupting effects (**Figure 8**).

## Conclusion

Several experimental strategies have been performed to demonstrate that new therapeutic approaches may have beneficial effects on sperm quantity and quality in humans during or after heavy metals exposure, in agreement with a previous report (Wong and Cheng, 2011). In addition, the protective effects on testis structure including BTB integrity resulted for CdCl_2_ + PDRN animals also indicate that the used polynucleotidic mixture represents a potential adjuvant in the therapy of testicular functionality impairments.

## Author Contributions

Conceived and designed the experiments: LM, AM, and FS. Performed the experiments: HM, AB, and GaP. Contributed reagents/materials/analysis tools: NI and GiP. Analyzed the data: MR, DP, AP, CL, AV, and AG. Wrote the paper: LM, DP, DA, FS, and RL. All authors contributed to and approved the final draft of the manuscript.

## Conflict of Interest Statement

Author LM, FS, and AB are co-inventor on a patent describing therapeutic polydeoxyribonucleotide activity in testicular injury by torsion. Author FS has received research support from Mastelli for work on polydeoxyribonucleotide. Authors FS, AB, and DA are co-inventors on a patent describing therapeutic polydeoxyribonucleotide activity in chronic intestinal disease. The remaining authors declare that the research was conducted in the absence of any commercial or financial relationships that could be construed as a potential conflict of interest.

## Abbreviations

AJ: adherens junction
BTB: blood-testis barrier
Cd: Cadmium
DAB: Diaminobenzidine
EDTA: ethylenediamine-tetraacetic acid
EGTA: ethylene glycol tetraacetic acid
ELISA: Enzyme Linked Immune Absorbent Assay
FSH: Follicle-stimulating hormone
LH: luteinizing hormone
PDRN: Polydeoxyribonucleotide
pERK 1/2: phospho extracellular signal-regulated kinase
TGF- 3: transforming growth factor- 3
TJ: tight junction
TE: testosterone
