# Corrigendum: Polydeoxyribonucleotide, an adenosine-A2_A_ receptor agonist, preserves blood testis barrier from cadmium-induced injury

**DOI:** 10.3389/fphar.2022.1073543

**Published:** 2022-11-16

**Authors:** Francesco Squadrito, Antonio Micali, Mariagrazia Rinaldi, Natasha Irrera, Herbert Marini, Domenico Puzzolo, Antonina Pisani, Cesare Lorenzini, Andrea Valenti, Rosaria Laurà, Antonino Germanà, Alessandra Bitto, Gabriele Pizzino, Giovanni Pallio, Domenica Altavilla, Letteria Minutoli

**Affiliations:** ^1^ Department of Clinical and Experimental Medicine, University of Messina, Messina, Italy; ^2^ Department of Biomedical and Dental Sciences and Morphofunctional Imaging, University of Messina, Messina, Italy; ^3^ Department of Human Pathology, University of Messina, Messina, Italy; ^4^ Department of Veterinary Sciences, University of Messina, Messina, Italy

**Keywords:** cadmium, PDRN, blood-testis barrier, TGF-β3, pERK 1/2, TUNEL, immunohistochemistry, transmission electron microscopy

In the published article, the Conflict of Interest statement was incorrectly filed. The correct Conflict of Interest statement appears below.

“Author LM, FS, and AB are co-inventor on a patent describing therapeutic polydeoxyribonucleotide activity in testicular injury by torsion. Author FS has received research support from Mastelli for work on polydeoxyribonucleotide. Authors FS, AB, and DA are co-inventors on a patent describing therapeutic polydeoxyribonucleotide activity in chronic intestinal disease.

The remaining authors declare that the research was conducted in the absence of any commercial or financial relationships that could be construed as a potential conflict of interest.”

The authors apologize for this error and state that this does not change the scientific conclusions of the article in any way. The original article has been updated.

